# Prognostic value of TOP2A in bladder urothelial carcinoma and potential molecular mechanisms

**DOI:** 10.1186/s12885-019-5814-y

**Published:** 2019-06-19

**Authors:** Shuxiong Zeng, Anwei Liu, Lihe Dai, Xiaowen Yu, Zhensheng Zhang, Qiao Xiong, Jun Yang, Fei Liu, Jinshan Xu, Yongping Xue, Yinghao Sun, Chuanliang Xu

**Affiliations:** 1 Department of Urology, Changhai Hospital, Second Military Medical University, Shanghai, People’s Republic of China; 2 Department of Geriatrics, Changhai Hospital, Second Military Medical University, Shanghai, People’s Republic of China

**Keywords:** Bladder urothelial carcinoma, TOP2A, Prognosis, Biomarker

## Abstract

**Background:**

The prognosis of bladder urothelial carcinoma (BLCA) varies greatly among patients, and conventional pathological predictors are generally inadequate and often inaccurate to predict the heterogeneous behavior of BLCA. This study aims to investigate the prognostic value and function of TOP2A in BLCA.

**Methods:**

TOP2A expression level was examined by RNA-sequencing, quantitative real time polymerase chain reaction and immunohistochemistry from 10, 40 and 209 BLCA samples, respectively. Public databases were analyzed for validation. Cell proliferation, migration, invasion assays were performed to explore potential functions of TOP2A in BLCA. Flow cytometry was performed for cell cycle and apoptosis analysis. Univariable and multivariable Cox regression models were performed to identify independent risk factors for the prognosis of BLCA.

**Results:**

We found TOP2A was significantly upregulated in BLCA samples, especially for high-grade and advanced stage tumors, compared with matched normal epithelial tissue. Univariable COX regression analysis revealed high TOP2A expression was significantly associated with poorer cancer-specific, progression-free and recurrence-free survival, but not independently of clinical characteristics in the multivariable models. Knockdown of TOP2A remarkably inhibited the proliferation of BLCA cells and non-cancerous urothelial cells. Furthermore, migration and invasion capacity of BLCA cells were strongly suppressed after TOP2A knockdown. Moreover, flow cytometry suggested TOP2A had anti-apoptotic function, and knockdown of TOP2A could induce resistance to doxorubicin in J82 cells.

**Conclusions:**

In our study, TOP2A was overexpressed in BLCA and could serve as a prognostic biomarker for BLCA. Moreover, TOP2A is functionally important for the proliferation, invasion and survival of BLCA cells.

**Electronic supplementary material:**

The online version of this article (10.1186/s12885-019-5814-y) contains supplementary material, which is available to authorized users.

## Background

Bladder urothelial carcinoma (BLCA) is the most common neoplasm in the urological system in China [1]. Five to 10 % of patients are already at metastatic stage at diagnosis, and around 50% of patients will develop local or distant disease progression after radical cystectomy [2]. Conventional pathological predictors such as tumor stage and grade are generally inadequate and often inaccurate to predict the heterogeneous behavior of BLCA [3]. Identification of recurrent genetic alteration in BLCA is critical for discovering genes that drive BLCA and identifying prognostic biomarkers for stratification of patients with different risks of prognosis [4]. With the advent of next-generation sequencing technology and computational biology, the understanding of BLCA has entered a new era [4]. Gaining insight into the biology of bladder cancer may reveal numerous biomarkers that enhance the sensitivity and specificity of early diagnosis and treatment of BLCA [5].

Topoisomerase-II alpha (TOP2A) is an essential nuclear enzyme which regulates the topological state of DNA during transcription and is involved in the processes of chromosome condensation and chromatid separation [6]. Several studies have reported that higher expression levels of TOP2A are indicative of poor prognosis in a variety of human cancers [6–9]. Moreover, TOP2A is the target for some of the most widely used chemotherapeutic drugs for treatment of human cancers [10, 11]. Despite the well-known association between TOP2A expression and aggressiveness of different cancers, the underlying role of TOP2A in the BLCA remains unclear.

In the present study, we explored the expression and function of TOP2A in BLCA patient samples and cell lines. We found that TOP2A was overexpressed in BLCA, and higher TOP2A expression was associated with poorer cancer-specific, progression-free and recurrence-free survival. We also revealed that TOP2A regulated proliferation and invasion, and played a role associated with anti-apoptosis in BLCA.

## Methods

### Clinical samples and cell lines

Ten pairs of BLCA samples (five muscle-invasive bladder cancer (MIBC) and five non-muscle invasive bladder cancer (NMIBC), eight high-grade and two low-grade) and their matched adjacent normal epithelial tissue were collected for RNA-sequencing (RNA-seq) from patients who were treated with cystectomy from June 2013 to May 2014. Another 40 BLCA samples (21 MIBC and 19 NMIBC, 26 high-grade and 14 low-grade) were collected from transurethral resection of bladder cancer (TURBT) or cystectomy for further validation by real time quantitative polymerase chain reaction (RT-qPCR). A total of 209 formalin-fixed BLCA tumors (130 MIBC and 79 NMIBC, 158 high-grade and 51 low-grade, median age 69 years, 144 males and 65 females, 144 from cystectomy and 65 from TURBT) were collected for immunohistochemistry from July 2008 to December 2015. No patient received chemotherapy or radiotherapy previously in this study. All clinical samples were obtained after written informed consents were provided by the patients, and the study protocol was approved by the Ethics Committee of Changhai Hospital. BLCA cells lines used in this study were purchased from the American Type Culture Collection (Manassas, VA, U.S.) and cells were cultured with the recommended medium and condition. Gemcitabine (Catalog No.S1149) and doxorubicin (Catalog No.S1208) was purchased from Selleck.

### Gene expression analysis by RNA-sequencing

RNAs were purified from tumor tissue for RNA-Seq analysis by Beijing Genomics Institute (Shenzhen, China). All samples were examined by pathologists to ensure that tumor sample had tumor density > 80% and the adjacent normal tissue were without tumor contamination. Experimental procedures of RNA-Seq were described previously [12, 13].

### Database mining

Public databases: gene expression omnibus (GEO, GSE31684, *n* = 93) (http://www.ncbi.nlm.nih.gov/gds/), and two comprehensive studies, i.e. The Cancer Genome Atlas (TCGA, bladder cancer, *n* = 413) and MSKCC (bladder cancer, *n* = 97) on BLCA gene expression profile and prognosis in cBioPortal (http://www.cbioportal.org/) were included for data mining.

### RNA extraction and RT-qPCR

Total RNA was extracted by TRIzol reagent (Invitrogen™), and the cDNA was synthesized using a PrimeScript RT Reagent kit (Takara, Japan) following the manufacturer’s instruction. RT-qPCR was conducted on an Applied Biosystems Step One Plus (Agilent, USA) using SYBR® Premix Ex Taq™ (Takara, Japan). The relative fold changes were calculated with 2 − ΔΔCT methods. The primers used are as follows: 5′-CATTGAAGACGCTTCGTTATGG-3′ (forward) and 5′- CCAGTTGTGATGGATAAAATTAATCAG-3′ (reverse) for TOP2A gene; 5′-ACGGTAGCTGCTAAAAAGGGAA-3′ (forward) and 5′- GGATGTTTTCCTGCCAGGGT-3′ (reverse) for TOP2B; 5′- ACCACAGTCCATGCCATCAC-3′ (forward), 5′- TCCACCACCCTGTTGCTGTA-3′ (reverse) for GAPDH.

### Immunohistochemistry

Formalin-fixed paraffin-embedded BLCA samples were cut into 5-μm-thick sections. Primary antibody (1:300) anti-TOP2A (ab52934) and anti-Ki67 (ab15580) was purchased from Abcam®. Immunohistochemistry staining was conducted according to the instruction of the immunohistochemistry kit (BioGenex, Fremont, CA, U.S.). The immunohistochemical stain of TOP2A was evaluated by pathologists in a blinded fashion and was scored on the percentage of positive tumor cell nucleus (negative, score 0; < 1/3, score 1; 1/3–2/3, score 2; > 2/3, score 3). Scores of 0–1 were defined as low expression, and 2–3 were defined as high expression.

### RNA interference and lentivirus construction

The small interference RNA (siRNA) against TOP2A (5′- GGUCAGAAGAGCAUAUGAUTT-3′; antisense, 5′- AUCAUAUGCUCUUCUGACCTT-3′ for siRNA-1; sense, 5′-GACCAACCUUCAACUAUCUTT-3′; antisense, 5′- AGAUAGUUGAAGGUUGGUCTT-3′ for siRNA-2; sense, 5′-GCTGCGGACAACAAACAAATT − 3′; antisense, 5′- GCTATCAGCCTGGCCTTTATT-3′ for siRNA-3) and non-specific siRNA (sense 5′-UUCUCCGAACGUGUCACGUTT-3′, antisense 5′-ACGUGACACGUUCGGAGAATT − 3′) were purchased from GenePharma, Shanghai, China. Cells were transfected with siRNA according to the instruction of the manufacturer (Lipofectamine® RNAiMAX, Invitrogen). Lentivirus with small hairpin RNA against TOP2A (5′- GATCCGGTCAGAAGAGCATATGATTTCAAGAGAATCATATGCTCTTCTGACCTTTTTTC-3′) and non-specific small hairpin RNA (5′- CTAGCCCGGCCAAGGAAGTGCAATTGCATACTCGAGTATGCAATTGCACTTCCTTGGTTTTTTGTTAAT-3′) were purchased from Hanbio (Shanghai, China). J82 Cells were transfected with lentiviral shRNA at a multiplicity of infection of about 30 in the presence of 5 μg/ml polybrene. Puromycin was added to medium at the concentration of 2.5 mg/ml for stable cell line selection.

### Cell proliferation, wound-healing and Matrigel invasion assays

Cell proliferation and drug sensitivity assay was conducted per the instruction of Cell Counting Kit-8 (CCK-8; Dojindo). Cells with a density of 3 × 10^3^ per well were seeded in triplicates in 96-well plates with 200 μL medium, and cell viability was determined using CCK-8 kit by adding 20 μL reagent. The cells were incubated for another 2 h and then the absorbance at 495 nm was measured with EnSpire Reader. Wound-healing assay was conducted by seeding cells in triplicate in 6-well plates. After the cell density reached about 80%, then 10 μL pipette tips were applied to create a cell-free trip area and the floating cells were washed away by PBS buffer. Cells were then cultured with pure medium without 10% fetal bovine serum (FBS). Photographs of the scratched area were taken at 0 h, 12 h, 24 h and 72 h, respectively. Invasion assays were performed with the transwell chamber with Matrigel (Millipore, USA). Cells with a density of 5 × 10^4^ were seeded into the upper chamber and cultured with 500 μL pure medium without 10% FBS, and the lower chamber were added with 1 ml complete medium. After 24 h or 48 h, cells remaining on the upper membrane were removed with cotton wool, and then placed into 4% formalin for 20 min. The cells on the membrane were stained with 0.1% crystal violet for 30 min. Microscope photographs of 10 random filed of the membrane were taken, and cells were counted for further statistical analyses.

### Tumor formation in nude mice

All animal experiments were conducted with the approval of the Scientific Investigation Board of the Second Military Medical University. J82 cells infected with lentivirus against TOP2A and non-specific lentivirus were injected subcutaneously into the lower flank of female athymic BALB/c nude mice. Each group had 6 mice and each mouse was injected with 1*10^6^ J82 cells. Tumor size was measured weekly and tumor volume was calculated using the following formula: Volume = Length x width^2^x 0.5(mm^3^). Mice with tumor diameter > 1.5 cm or with losses in body weight of > 20% were promptly euthanized by asphyxiation by CO2 inhalation from compressed gas source. All animal studies were performed according to the Guidelines of the Second Military Medical University for the Care and Use of Laboratory Animals.

### Flow cytometry analysis

Cells were collected for flow cytometry analysis 72 h after transfection with TOP2A siRNA or control siRNA. Cells were stained using cell cycle kit (Lianke Biotech Co., Ltd., China) per manufacturer’s instructions, and then analyzed on a Beckman Coulter Analyzer (Beckman Coulter, Brea, CA, USA). Cell cycle data were analyzed with Modfit software. Apoptosis was performed using Annexin V fluorescein isothiocyanate and propidium iodide apoptosis kit (Lianke Biotech Co., Ltd., China). Stained cells were analyzed on a MACSQuant Analyzer (Teterow, Germany), and data was then analyzed by FlowJo v7.6.1 software.

### Statistics

At least three independent experiments were performed for each experiment described in this study. Student *t* test or one-way ANOVA was used to compare continuous parametric data between two groups and multiple groups, respectively. Kaplan-Meier survival curve and log-rank test were applied for survival analysis. Univariable and multivariable Cox proportional hazards models were performed to identify independent risk factors for the prognosis of BLCA. A backward step-down wald selection method was used (the entry with *P* < 0.05 and removal criteria with *P* < 0.10). The statistical analysis was performed either by SPSS 19.0 (IBM Inc.) or GraphPad Prism 5 (GraphPad Software, Inc., La Jolla, CA).

## Results

### TOP2A was up-regulated in BLCA

**Fig. 1:**
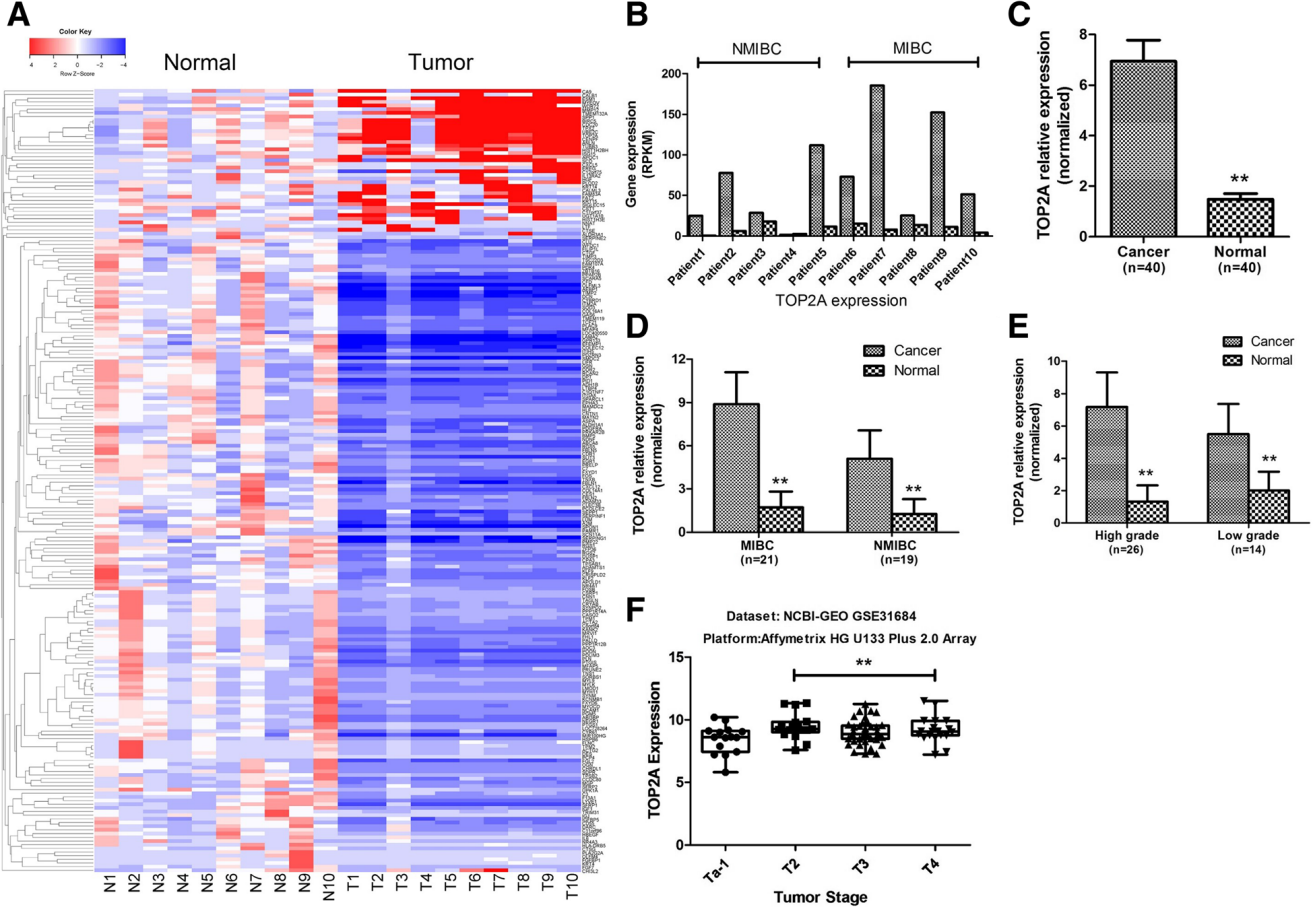
Increased TOP2A expression in bladder urothelial carcinoma (BLCA) revealed by RNA-sequencing (**a**) and RT-qPCR (**c**-**e**). **a** Hierarchical clustering heat map of differentially expressed genes between tumor tissue and matched normal epithelial tissue. **b** Expression levels of TOP2A in each sample for RNA-sequencing. **c** TOP2A gene expression was significantly up regulated in tumor tissue compared with matched normal epithelial tissue. **d** TOP2A gene expression in different stages of BLCA. **e** TOP2A gene expression in different grades of BLCA. **f** TOP2A gene expression was significantly higher in MIBC compared with NMIBC in NCBI-GEO GSE31684 dataset. MIBC = muscle-invasive bladder cancer; NMIBC = non-muscle invasive bladder cancer; RPKM = reads per gene per kilobase exon per million mapped reads. **, *p* < 0.01

We performed RNA-Seq on ten pairs of matched BLCA tumor samples and the adjacent normal tissue obtained by cystectomy. We used a cutoff of log2-fold change > 2 and probability > 0.8, a method proposed by Tarazona et al. [14], to filter differentially expressed genes between tumor samples and the matched normal epithelial tissue (Fig. 1a). We identified TOP2A was up-regulated in nine out of ten tumor samples with a log2-fold change of 3.02 and probability of 0.86 (Fig. 1b). As shown in Fig. 1b, the average level of TOP2A, represented by reads per gene per kilobase exon per million mapped reads (RPKM), was higher in MIBC than NMIBC. We further validated the expression of TOP2A in an additional 40 tumor samples and the matched normal epithelial tissue using RT-qPCR, the baseline characteristics of the patients were shown in Table 1. We found that TOP2A was significantly up-regulated in tumor samples (Fig. 1c), especially higher for MIBC (Fig. 1d) and high-grade tumors (Fig. 1e). We further explored the NCBI-GEO datasets to determine the expression levels of TOP2A in BLCA. We found that the TOP2A expression was significantly higher in MIBC compared with NMIBC in GSE31684 dataset, which included 93 patients with high-risk BLCA from Memorial Sloan-Kettering Cancer Center (MSKCC, Fig. 1f).

**Table 1:** Baseline characteristics of patients for RT-qPCR and IHC validation

	RT-qPCR cohort (*n* = 40)	IHC cohort (*n* = 209)
Age, median(IQR)	65 (56,70)	69 (61, 75)
Gender, men/women	31/9	144/65
Surgery type		
TURBT	12	65
Cystectomy	28	144
Pathological stage		
Ta, Cis, T1	19	79
T2	13	70
T3	6	54
T4	2	6
Grade		
Low	14	51
High	26	158
Lymph node status		
negative	38	198
positive	2	11

RT-qPCR = real time quantitative polymerase chain reaction; IHC = immunohistochemistry; TURBT = transurethral resection of bladder tumor; IQR = Interquartile range

### TOP2A expression correlated with prognosis of BLCA

**Fig. 2:**
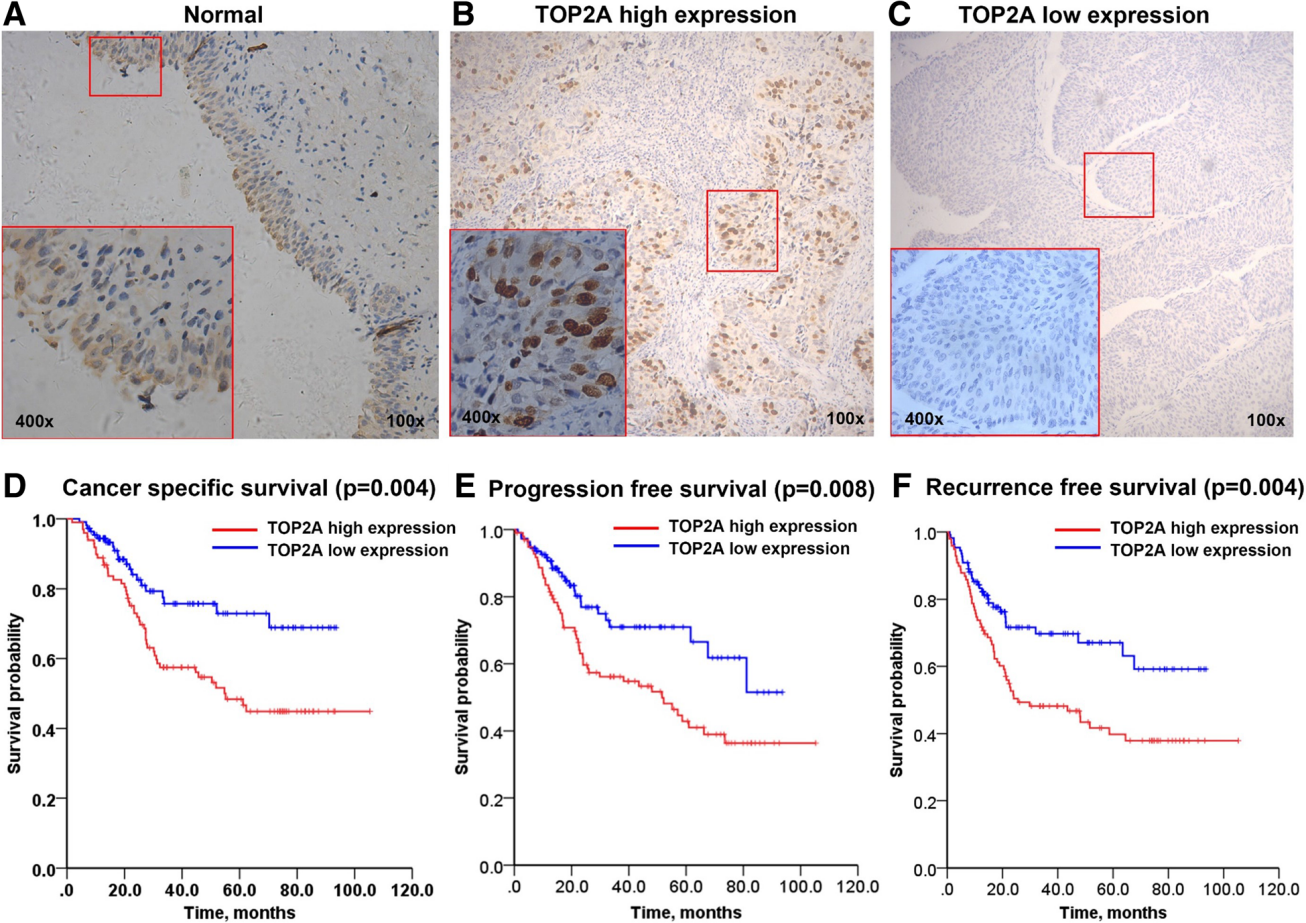
The relationship between TOP2A protein expression and prognosis of patients with BLCA. **a-c** Representative images of TOP2A protein expression in normal and bladder cancer tissue. **d-f** Higher TOP2A protein expression was associated with poorer cancer-specific, progression-free and recurrence-free survival from our single institution cohort (*n* = 209)

We evaluated the expression of TOP2A by immunohistochemical analysis in 209 patients who underwent transurethral resection of BLCA or cystectomy. The baseline characteristics of the patients were shown in Table 1. The TOP2A showed a totally nuclear expression pattern, and it was almost negative in all normal urothelium that could be studied adjacent to tumor tissue (Fig. 2a). Of the 209 tumor samples, high (staining score, 2.23 ± 0.42, Fig. 2b) and low (staining score, 0.95 ± 0.22, Fig. 2c) TOP2A expression was found in 99 and 110 patients, respectively. The TOP2A staining score was also significantly higher in high-grade tumor (1.67 ± 0.74) and MIBC tumor (1.56 ± 0.49) than low- grade tumor (1.11 ± 0.43, *P* < 0.001) and NMIBC tumor (1.27 ± 0.45, *P* < 0.001), respectively.

With a median follow-up of 27.9 months (range, 10 to 95 months), patients with high TOP2A expression showed significantly poorer cancer-specific survival (CSS, Fig. 2d, median, 21.3 vs. 41.1 months, *P* = 0.004), progression-free survival (PFS, Fig. 2e**,** median, 16.8 vs. 33.6 months, *P* = 0.008) and recurrence-free survival (RFS, Fig. 2f**,** 11.6 vs. 37.3 months, *P* = 0.004) compared with patients with low TOP2A expression. Survival analysis was further investigated on MIBC (*n* = 130) and NMIBC (*n* = 79) cohorts respectively. In MIBC cohort, patients with elevated TOP2A expression had significantly poorer CSS (*P* = 0.044), PFS (*P* = 0.049) and RFS (*P* = 0.041) compared with patients with low expression (Additional file 1: Figure S1). However, no difference was noticed between TOP2A high expression and low expression groups in NMIBC cohort (Additional file 1: Figure S1). These results suggested that TOP2A had better prognostic value for patients with MIBC. Detailed results of univariable and multivariable Cox regression analysis of predicative variables of CSS were summarized in Table 2. The level of TOP2A was revealed to be associated with CSS in univariable COX regression analysis. However, multivariable COX regression analysis showed tumor stage and lymph node status were independent predictors of CSS. We further revealed that TOP2A expression ran in parallel to Ki67, which was an established proliferation biomarker, with a correlation coefficient of 0.6 (Spearman test with *P* < 0.01).

**Table 2:** Univariable and multivariable COX regression models for predicting cancer specific survival of bladder cancer

Variables	Univariable			Multivariable		
	HR	95% CI	*P* value	HR	95% CI	*P* value
Age (< 65 yr. as reference)						
≥ 65 yr	0.79	0.48–1.29	0.34			
Gender (female as reference)						
Male	1.07	0.63–1.80	0.81			
Pathological stage (T1 as reference)						
T2	2.22	1.13–4.36	0.02	1.67	0.83–3.37	0.15
T3	4.88	2.49–9.56	< 0.01	3.32	1.62–6.80	< 0.01
T4	22.45	8.09–62.30	< 0.01	16.70	5.85–47.69	< 0.01
Pathological grade (low as reference)						
High	2.33	1.19–4.56	0.01	1.82	0.89–3.72	0.10
Lymph node (negative or NA as reference)						
Positive	6.14	4.17–9.05	< 0.01	4.28	2.83–6.24	< 0.01
TOP2A expression (low as reference)						
High	2.09	1.25–3.49	< 0.01	1.42	0.81–2.46	0.21
Ki67 expression (low as reference)						
High	2.98	1.53–5.88	< 0.01	2.01	0.89–4.60	0.10

### The expression of TOP2A mRNA and protein diverged from each other

**Fig. 3:**
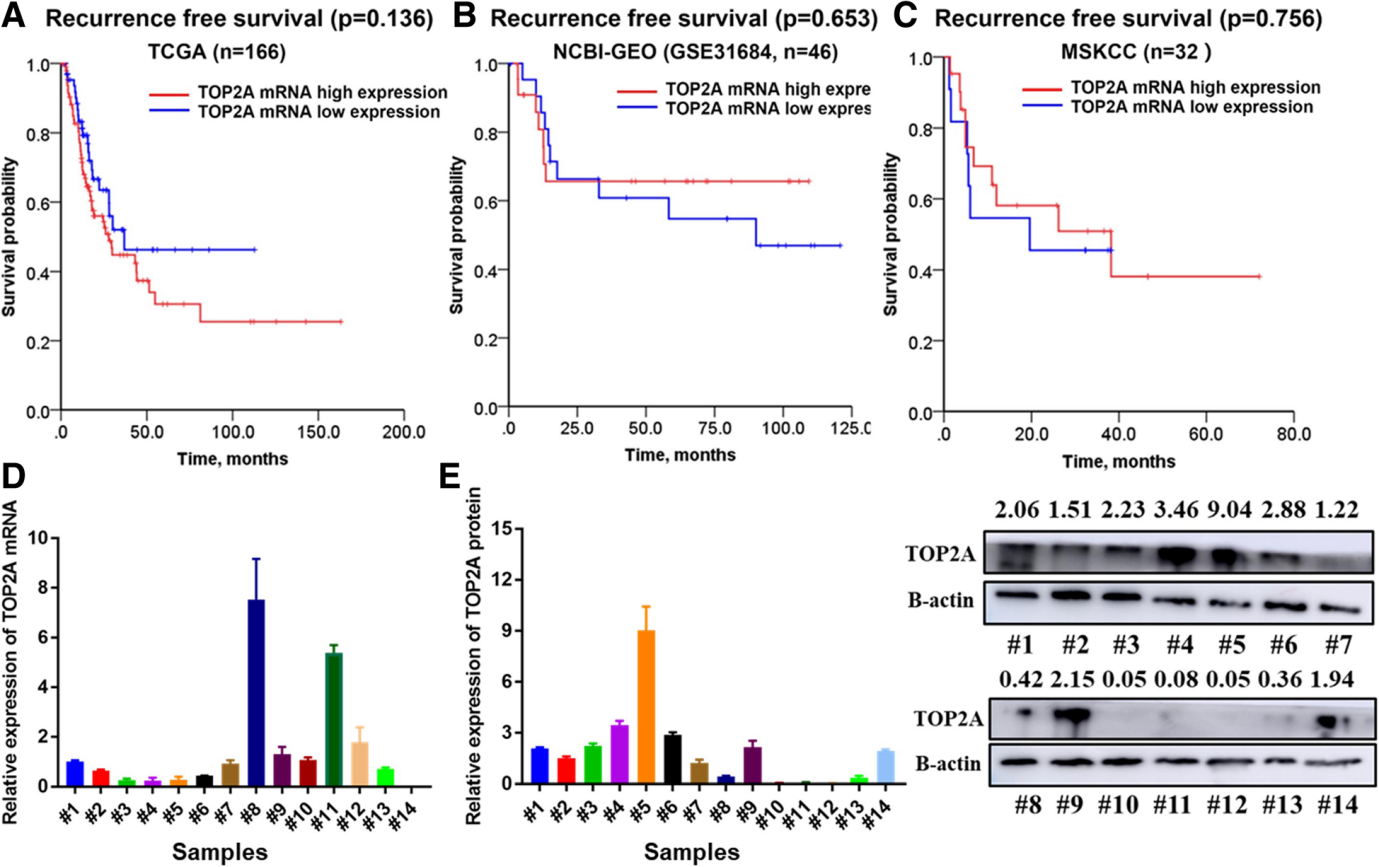
The prognostic value of TOP2A mRNA and its relationship with TOP2A protein in BLCA. **a-c** Kaplan-Meier survival curve comparing recurrence-free survival between patients with TOP2A mRNA high and low expression in TCGA, NCBI-GEO (GSE31684) and MSKCC datasets, respectively. Patients with TOP2A mRNA expression above 75% or below 25% were included for analysis. **d** RT-qPCR assay of TOP2A mRNA expression level in 14 bladder cancer samples. **e** TOP2A protein level of the 14 bladder cancer samples were investigated by western blotting assay

We further investigated the association between the levels of TOP2A mRNA and clinical outcome of patients in public databases of TCGA and NCBI-GEO. Patients with TOP2A mRNA expression above 75% (high expression) and below 25% (low expression) were included for further analysis [15]. As shown in Fig. 3a, patients with up-regulated TOP2A mRNA showed tendentially shorter recurrence-free survival compared to patients with low expression (27.8 vs. 36.8 months), but it did not reach statistical significance (*P* = 0.135). Regarding the NCBI-GEO (GSE31684, Fig. 3b) and MSKCC (bladder cancer, JCO, 2013, Fig. 2c) datasets, no significant differences were detected between TOP2A mRNA high expression and low expression groups. The expression pattern of TOP2A mRNA and protein was further investigated in 14 BLCA samples by RT-qPCR and western blot, respectively (Fig. 3d and e). We found no association between TOP2A mRNA and protein, with correlation coefficient of − 0.40 (Additional file 2: Figure S2, *P* = 0.15).

### Functional alteration of BLCA cells after knockdown of TOP2A

**Fig. 4:**
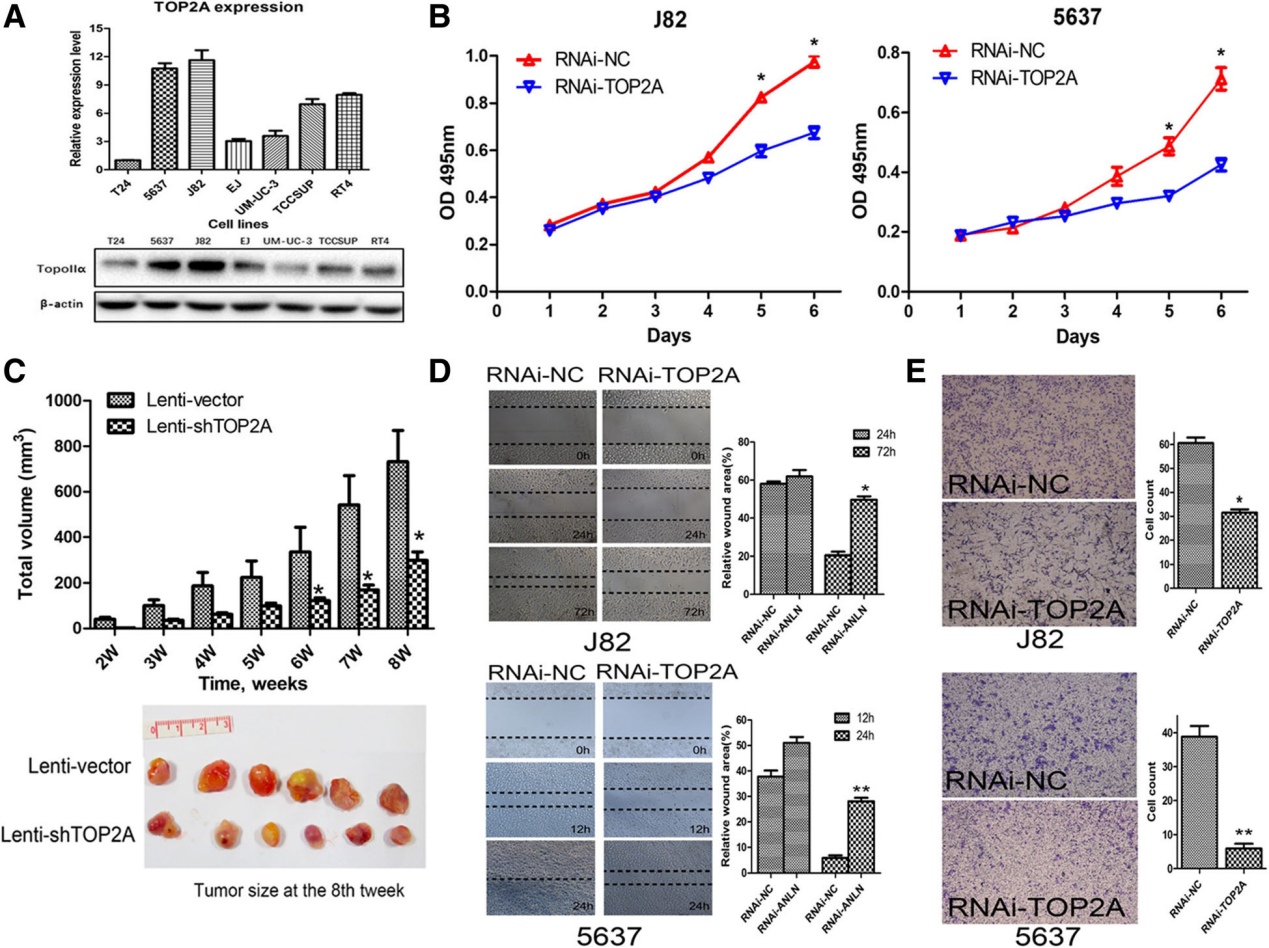
Cell proliferation, migration and invasion capacity was inhibited after TOP2A knockdown in bladder cancer cells. **a** TOP2A mRNA and protein expression levels in different bladder cancer cell lines. **b** The growth curves of J82 and 5637 cells between control and TOP2A knockdown groups. **c** The effect of TOP2A knockdown on tumor growth in nude mouse xenograft models. **d** Knockdown of TOP2A significantly decreased cell migration capacity in wound-healing assay. **e** Knockdown of TOP2A significantly reduced cells invading through Matrigel.NC = negative control. *, *p* < 0.05, **, *p* < 0.01. At least three independent replicates were performed for each experiment

We investigated the level of TOP2A in several bladder cancer cell lines, J82 and 5637 cells had relative higher TOP2A expression (Fig. 4a). To explore the potential function of TOP2A in BLCA, we knocked down TOP2A expression in J82 and 5637 cells using siRNA. The level of TOP2A mRNA and protein were significantly down-regulated after siRNA transfection in J82 and 5637 cells, while TOP2B expression was not influenced (Additional file 2: Figure S2). We then analyzed the proliferation rate of J82, 5637 and non-cancerous urothelial cells SVHUC after TOP2A knockdown. As shown in Fig. 4b, cell proliferation rate was significantly inhibited 5 days after TOP2A knockdown in J82 and 5637 cells. Meanwhile, we also observed that cell proliferation rate of SVHUC decreased after TOP2A knockdown (Additional file 2: Figure S2). Additionally, we developed J82 cells with TOP2A stably knocked down using lentivirus-mediated gene transfer method (Additional file 2: Figure S2). We further tested the effect of knockdown of TOP2A expression on growth as subcutaneous xenograft nude mice model. Tumor volume was measured from two to 8 weeks after J82 cells implantation. The difference of tumor volume between TOP2A knockdown group and control group was statistically significant 6 weeks after tumor cells implantation (Fig. 4c and Additional file 4: Table S1).

We also assessed the potential effect of TOP2A knockdown on bladder cancer cell migration and invasion, which were two critical steps accounted for cancer progression and metastasis. Wound healing assay showed that J82 and 5637 transfected with TOP2A siRNA displayed significantly lower migration capacity compared with control groups (Fig. **4**d). Moreover, the transwell Matrigel invasion assay also indicated that suppression of TOP2A expression strongly suppressed the invasion capacity of J82 and 5637 cells (Fig. 4e). These data suggested that TOP2A was closely associated with migration and invasion capacity of bladder cancer cells.

### Apoptosis and doxorubicin resistant induced by TOP2A knockdown

**Fig. 5:**
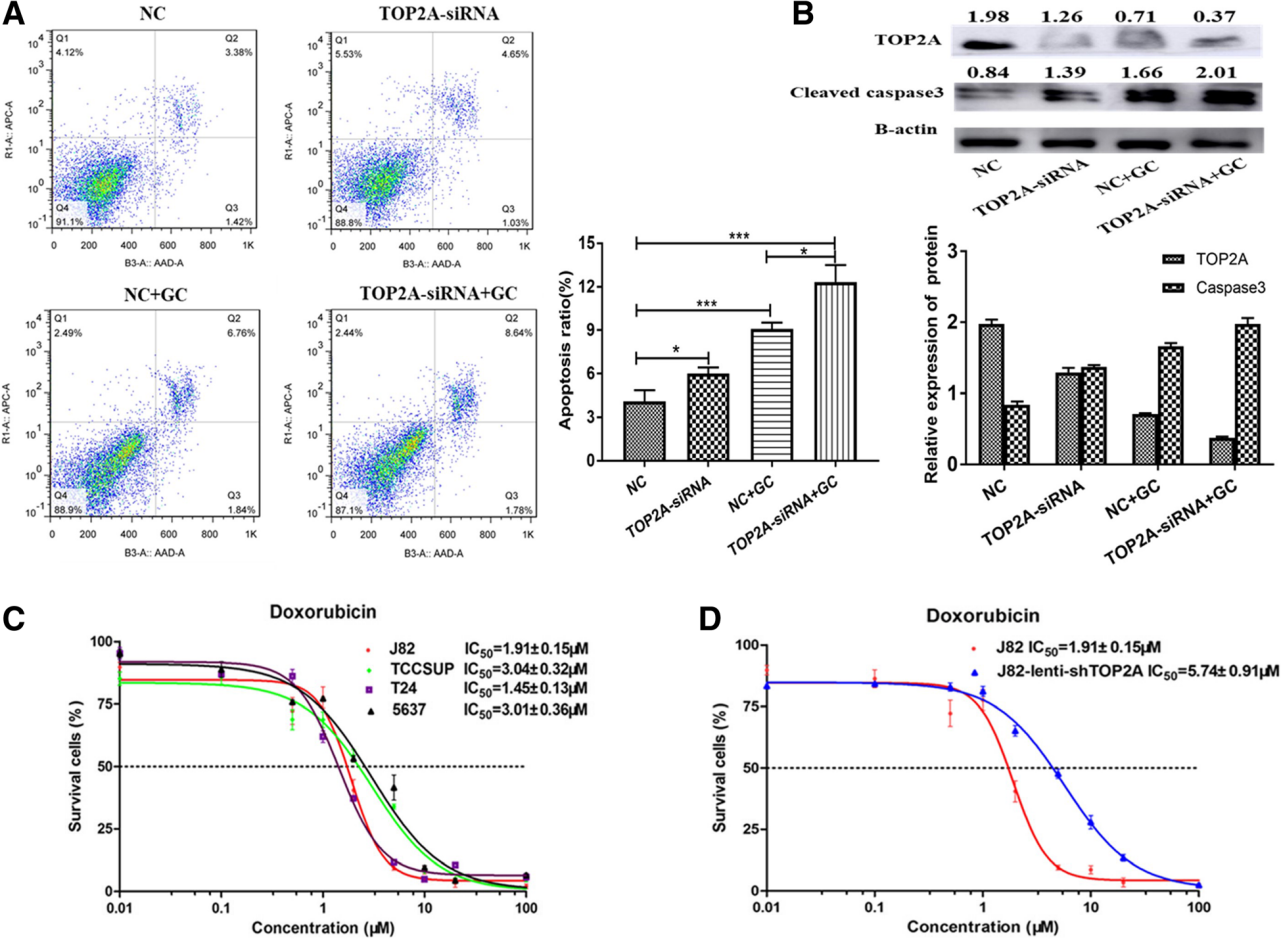
Apoptosis analysis after TOP2A knockdown. **a** Apoptosis were significantly increased after TOP2A knockdown in J82 and cells after induction of apoptosis by gemcitabine treatment for 24 h. **b** Western blotting assay revealed increasing expression of cleaved caspase 3 after TOP2A knockdown. **c-d** Dose response curves of bladder cancer cell lines treated with doxorubicin at different concentrations as determined in 72-h cell viability assays. NE = necrosis; NC = negative control; IC_50_ = half maximal inhibitory concentration *, *p* < 0.05. At least three independent replicates were performed for each experiment

We performed flow cytometry to compare cell cycle phases and apoptosis in J82 and 5637 cells between TOP2A knockdown and control cells. We found no significant changes in cell cycle phase distribution after TOP2A knockdown in J82 and 5637 cells (Additional file 3: Figure S3). In terms of apoptosis, knockdown of TOP2A showed a significant increase of early and late apoptosis in both J82 cells and cells after induction of apoptosis by gemcitabine treatment for 24 h (Fig. 5a). Western-blot assay further indicated the increasing expression of cleaved caspase 3 after TOP2A knockdown (Fig. 5b). These results suggested that TOP2A might play a role of anti-apoptosis in bladder cancer cells. TOP2A is the primary target of doxorubicin, which is an important chemotherapeutic drug for bladder cancer [2]. We thus explored whether TOP2A could serve as a marker of response to doxorubicin in bladder cancer. The sensitivity of TOP2A high expression (J82 and 5637) and low expression (T24 and TCCSUP) cell lines to doxorubicin was compared. According to the CCK-8 assay results (Fig. 5c), TOP2A expression level was not correlated with the sensitivity of bladder cancer cell lines to doxorubicin. Furthermore, we compared the sensitivity of J82 wild type cells (J82/WT) and J82 cells with TOP2A stably knockdown (J82/TOP2A) to doxorubicin. As shown in Fig. 5d, the half maximal inhibitory concentration (IC_50_) values for J82/WT and J82/TOP2A cells were 1.91 ± 0.15 μM and 5.74 ± 0.91 μM, respectively. J82 cells exhibited relatively resistance to doxorubicin after TOP2A knockdown.

## Discussion

Two types of topoisomerase II enzymes were expressed in mammalian cells, TOP2A and TOP2B, but only TOP2A plays a crucial role for cellular viability [10, 16]. TOP2A gene, mapped to chromosome 17q12-q21, encodes an enzyme which implicates in almost any processes of DNA metabolism including replication, transcription and chromosome segregation during interphase and mitosis [17, 18]. TOP2A is thus known to be a marker of cell proliferation in normal tissue, and increased TOP2A expression levels are observed in cancer cells compared with non-malignant cells [19, 20]. Previous studies also suggested that high TOP2A expression levels could indicate tumor aggressiveness and poor outcome [3, 6, 8, 21, 22].

In the present study, our RNA-Seq data of ten pairs of BLCA samples identified TOP2A gene were significantly upregulated in the tumor samples compared with the matched normal epithelial tissue. We further validated that the expression levels of TOP2A mRNA were elevated in tumors by RT-qPCR in a cohort of 40 samples. We found that TOP2A levels were higher in patients with MIBC and high-grade tumors compared with NMIBC and low-grade tumors, respectively. The results were in line with previous studies that TOP2A expression levels reflected the aggressiveness of tumors [3, 6, 8, 21, 22]. We further demonstrated that there were significant differences between patients with low- and high-expression of TOP2A protein in terms of CSS, PFS and RFS. However, we failed to find TOP2A as an independent risk factor for prognosis in the multivariable COX regression model, and tumor stage and lymph node status served as the most important prognostic factors for BLCA patients. It indicated that TOP2A was an important but not exclusive contributing factor for the progression of BLCA. This result also agreed with several previous studies in BLCA. Simon et al. [23] reported that TOP2A and HER-2 expression was co-amplified in BLCA, and TOP2A amplification were associated with advanced tumor stage and high grade, but they also found no independent prognostic value of TOP2A amplification or protein expression in multivariable COX regression analysis. Koren et al. [24] investigated the expression of TOP2A protein by immunohistochemistry in 57 specimens and found higher TOP2A expression indicated greater probability of BLCA recurrence and lower overall survival. Subgroup analysis of the prognostic value of TOP2A in MIBC and NMIBC in the present study further revealed that TOP2A had better prognostic value for patients with MIBC rather than NMIBC. This was in contrast to a previous study, Nakopoulou et al. [25] reported that higher TOP2A expression was also indicative of worse prognosis for patients with superficial bladder tumors (*n* = 51). Taken together, TOP2A is a promising biomarker for differentiating patients with different risks of BLCA, and combining TOP2A and conventional prognostic factors, such as tumor stage, may increase the accuracy of predicting prognostic outcomes and provide additional arguments for treatment and surveillance decisions.

Whether the level of TOP2A mRNA could serve as a predictor for the prognosis of BLCA is in controversy. In the present study, public databases analysis showed that patients with higher TOP2A mRNA expression did not significantly correlate with the prognosis. We also found no correlation between the level of TOP2A mRNA and protein. It suggests that further studies are warranted to explore the post-transcription mechanisms that contribute to interpret the divergent levels between TOP2A mRNA and protein in BLCA. Meanwhile, Ren et al. [17] revealed high TOP2A expression correlated with worse cancer prognosis, but no relationship between amplification of TOP2A gene and prognosis of cancer in the meta-analysis of 25 studies. In contrast, Kim et al. [3] reported that elevated expression of TOP2A mRNA was indicative of high rate of recurrence and progression in NMIBC (*n* = 103).

Although TOP2A alteration was closely associated with prognosis of BLCA, the oncogenic function of TOP2A in BLCA has not been studied previously. The biological behavior of cancers is generally influenced by two major biological processes: proliferation and invasion [25]. In the present study, we found that knockdown of TOP2A could significantly inhibit the proliferation of bladder cancer cells and non-cancerous urothelial cells, which revealed the essential role of TOP2A in cell proliferation. In addition, knockdown of TOP2A strongly suppressed the migration and invasion capacity of J82 and 5637 cells. Furthermore, flow cytometry analysis suggested that TOP2A played a role in anti-apoptosis in BLCA. These results strengthened the evidence that TOP2A involved in the progression of BLCA. Jain et al. [6] reported that TOP2A was overexpressed in adrenocortical carcinoma and might influence tumor progression, as knockdown of TOP2A in adrenocortical carcinoma cells decreased cell proliferation, and invasion. Gobble et al. [26] found TOP2A was overexpressed in liposarcoma, and knockdown of TOP2A in liposarcoma cell lines reduced proliferation, invasiveness and increased apoptosis. Taken together, TOP2A was proved to play a major role in proliferation and invasion.

Given the essential role of TOP2A in malignant tumors, TOP2A has held the interest of researchers developing targeted anticancer drugs [11]. Doxorubicin and etoposide are two most clinically active anticancer agents targeting TOP2A in different cancers [27–29]. Previous studies suggested that the levels of TOP2A expression could determine the response of chemotherapeutic drugs targeting this enzyme [10, 30, 31]. Tumors with low expression of TOP2A had fewer TOP2A mediated DNA strand breaks induced by targeted drugs and was thus less sensitive than tumors with high level of TOP2A [30]. In the present study, we showed that TOP2A knockdown induced doxorubicin resistance in J82 cells, however, the sensitivity of different bladder cancer cell lines to doxorubicin was not significantly correlated with the expression level of TOP2A. The results implied that the sensitivity of bladder cancer cell lines to doxorubicin might be determined by multiple factors. In addition to TOP2A down-regulation, AbuHammad et al. [32] revealed that metabolizing genes (specifically CYP1A1 and CYP1A2) and other genes were crucial for cell cycle, apoptosis and DNA repair were involved in the development of doxorubicin resistance to breast cancer cells. As a result, combinational biomarkers rather than sole TOP2A expression level warrants further investigation to predict the sensitivity of BLCA to doxorubicin.

## Conclusions

In summary, we identified that the expression of TOP2A was overexpressed in BLCA at both mRNA and protein levels, and TOP2A protein level could serve as a promising prognostic biomarker for BLCA. Furthermore, we found that TOP2A played a major role in BLCA by regulating proliferation, invasion and survival of bladder cancer cells.

## Additional files

Additional file 1: **Figure S1.** Kaplan-Meier survival curve comparing patients with different expression levels of TOP2A. A Cancer specific survival, progression free survival and recurrence free survival curve between TOP2A high and low expression patients with muscle invasive bladder cancer (MIBC, *n*=130). B Cancer specific survival, progression free survival and recurrence free survival curve between TOP2A high and low expression patients with non-muscle invasive bladder cancer (NMIBC) patients (*n*=79). (JPG 435 kb)

Additional file 2: **Figure S2.** The effect of TOP2A RNA interference on the expression of TOP2A in bladder cancer cells. A Pearson correlation analysis between TOP2A mRNA and protein in bladder cancer samples. B TOP2A was efficiently down-regulated by RNA interference (RNAi) in J82 and 5637 cells. C TOP2B mRNA expression was not influenced by RNA interference (RNAi) against TOP2A in J82 and 5637 cells. D The growth curves of non-cancerous urothelial cells SVHUC showed proliferation rate was inhibited 5 days after TOP2A knockdown. E TOP2A was efficiently down-regulated in J82 cells after transfection of lentivirus against TOP2A. (JPG 546 kb)

Additional file 3: **Figure S3.** The effect of TOP2A knockdown on the progression of cell cycle in bladder cancer cells. No significant difference was detected in cell cycle distribution after TOP2A knockdown in J82 and 5637 cells. (JPG 502 kb)

Additional file 4: **Table S1.** Tumor volumes of subcutaneous xenograft nude mice models in control group and TOP2A knockdown group. This table included the diameter and volume of the tumor sizes in control group and TOP2A knockdown group, which were measured as indicated in the methods section. (XLSX 10 kb)

## Data Availability

The datasets used and analyzed in the current study are available from the corresponding author on reasonable request.

## Abbreviations

BLCA: Bladder urothelial carcinoma
CSS: Cancer-specific survival
MIBC: Muscle-invasive bladder cancer
NMIBC: Non-muscle invasive bladder cancer
PFS: Progression-free survival
RFS: Recurrence-free survival
RNA-seq: RNA-sequencing
RPKM: Reads per gene per kilobase exon per million mapped reads
RT-qPCR: Real time quantitative polymerase chain reaction
TOP2A: Topoisomerase-II alpha
TURBT: Transurethral resection of bladder cancer
