# Supplementary figures and images for: Prognostic value of TOP2A in bladder urothelial carcinoma and potential molecular mechanisms
Source: BMC Cancer. 2019 Jun 19;19:604. doi: 10.1186/s12885-019-5814-y (PMC6582551; doi:10.1186/s12885-019-5814-y)

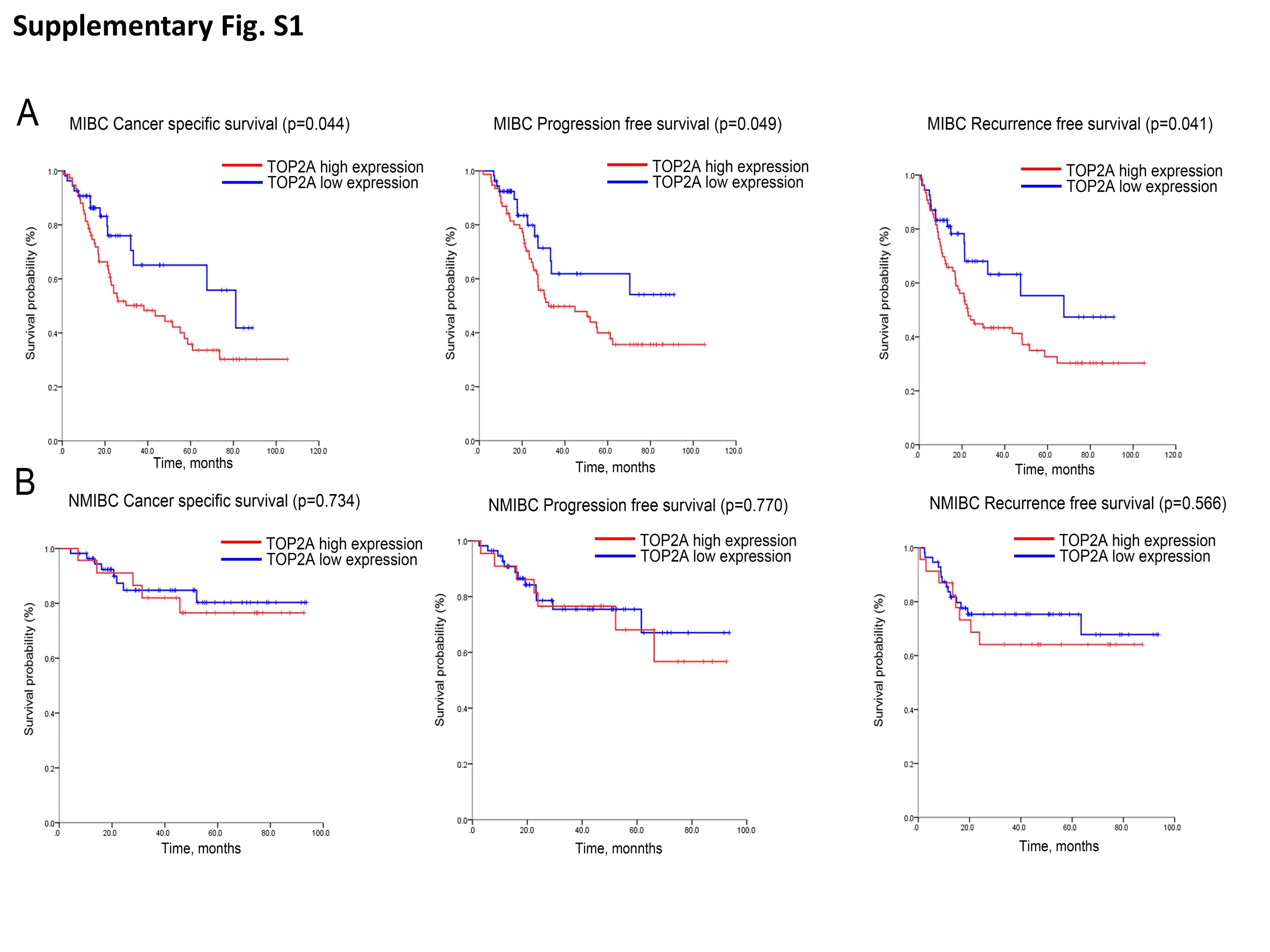

Supplement: Supplementary file 1 — Figure S1. Kaplan-Meier survival curve comparing patients with different expression levels of TOP2A. A Cancer specific survival, progression free survival and recurrence free survival curve between TOP2A high and low expression patients with muscle invasive bladder cancer (MIBC, n=130). B Cancer specific survival, progression free survival and recurrence free survival curve between TOP2A high and low expression patients with non-muscle invasive bladder cancer (NMIBC) patients (n=79). (JPG 435 kb) [file 12885_2019_5814_MOESM1_ESM.jpg]

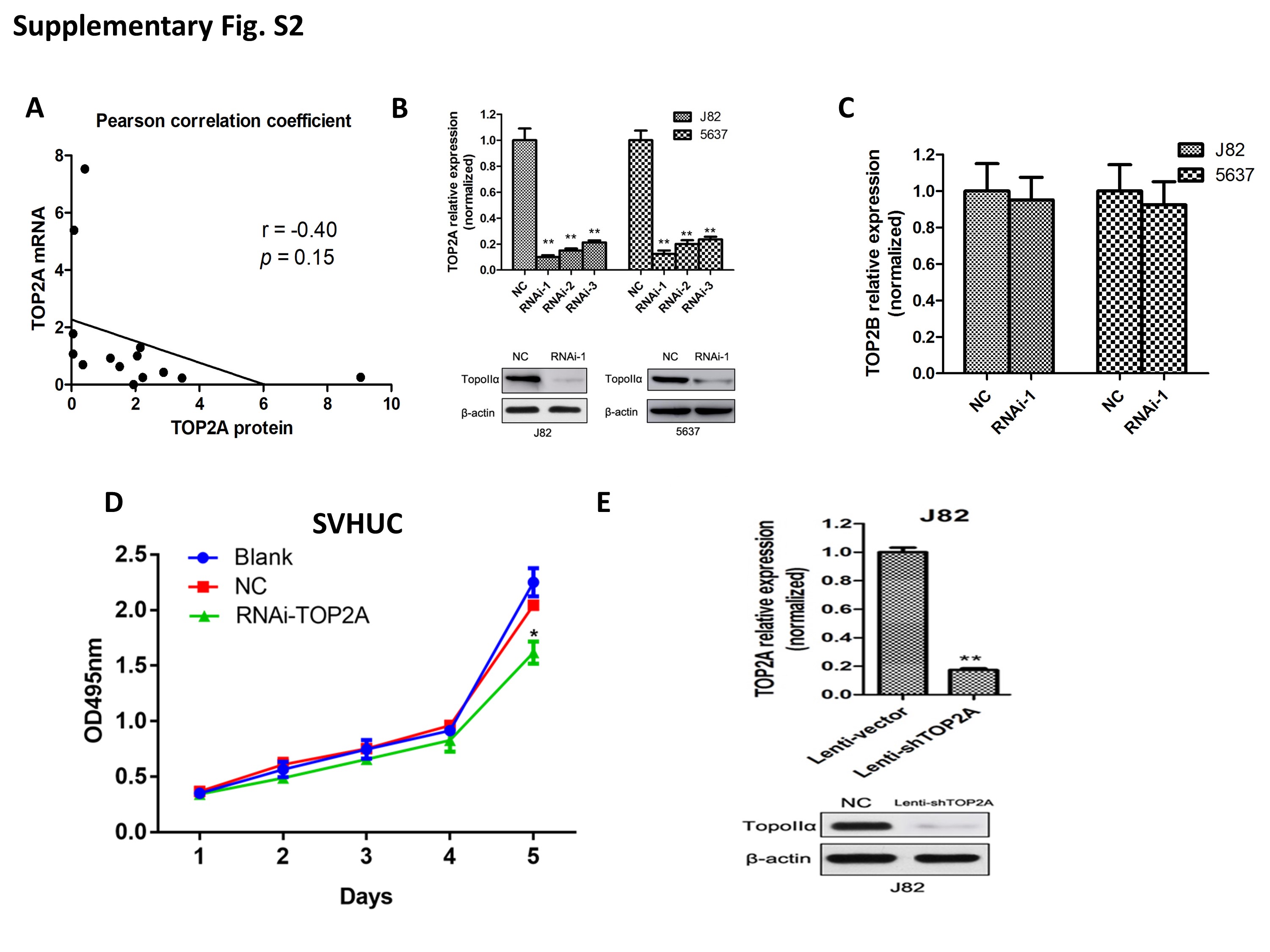

Supplement: Supplementary file 2 — Figure S2. The effect of TOP2A RNA interference on the expression of TOP2A in bladder cancer cells. A Pearson correlation analysis between TOP2A mRNA and protein in bladder cancer samples. B TOP2A was efficiently down-regulated by RNA interference (RNAi) in J82 and 5637 cells. C TOP2B mRNA expression was not influenced by RNA interference (RNAi) against TOP2A in J82 and 5637 cells. D The growth curves of non-cancerous urothelial cells SVHUC showed proliferation rate was inhibited 5 days after TOP2A knockdown. E TOP2A was efficiently down-regulated in J82 cells after transfection of lentivirus against TOP2A. (JPG 546 kb) [file 12885_2019_5814_MOESM2_ESM.jpg]

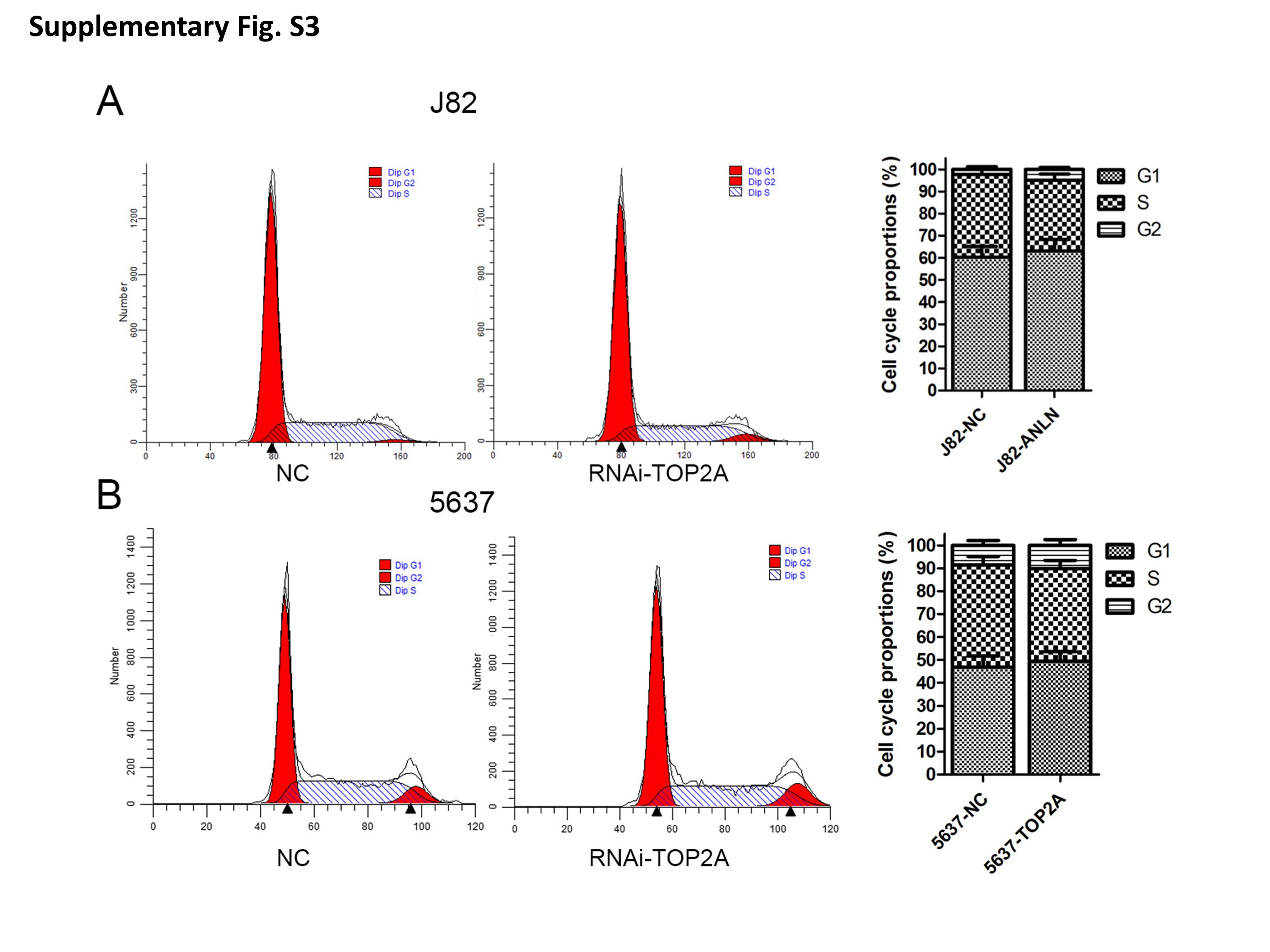

Supplement: Supplementary file 3 — Figure S3. The effect of TOP2A knockdown on the progression of cell cycle in bladder cancer cells. No significant difference was detected in cell cycle distribution after TOP2A knockdown in J82 and 5637 cells. (JPG 502 kb) [file 12885_2019_5814_MOESM3_ESM.jpg]
